# Synthesis of hapten and preparation of specific polyclonal antibody with high affinity for lenalidomide, the potent drug for treatment of multiple myeloma

**DOI:** 10.1186/1752-153X-6-125

**Published:** 2012-10-26

**Authors:** Ibrahim A Darwish, Nourh Z Alzoman, Reem M Abuhejail, Tilal E El-Samani

**Affiliations:** 1 Department of Pharmaceutical Chemistry, College of Pharmacy, King Saud University, P.O. Box 2457, Riyadh, 11451, Saudi Arabia

**Keywords:** Multiple myeloma, Lenalidomide, Polyclonal antibody, ELISA, Therapeutic monitoring, Pharmacokinetic studies

## Abstract

**Background:**

For therapeutic monitoring and pharmacokinetic studies of lenalidomide (LND), the potent drug for treatment of multiple myeloma (MM), a specific antibody was required for the development of a sensitive immunoassay system for the accurate determination of LND in plasma.

**Results:**

In this study, a hapten of LND (*N-*glutaryl*-*LND) was synthesized by introducing the glutaryl moiety, as a spacer, into the primary aromatic amine site of the LND molecular structure. The structure of the hapten (G-LND) was confirmed by mass, ^1^H-NMR, and ^13^C spectrometric techniques. G-LND was coupled to each of bovine serum albumin (BSA) and keyhole limpet hemocyanin (KLH) proteins by ethyl-3-(3-dimethylaminopropyl) carbodiimide as a coupling reagent. LND-KLH conjugate was used as an immunogen. Four female 2-3 months old New Zealand white rabbits were immunized with an emulsion of LND-KLH with Freund`s adjuvant. The immune response of the rabbits was monitored by direct enzyme-linked immunosorbent assay (ELISA) using LND-BSA immobilized onto microwell plates as a solid phase. The rabbit that showed the highest antibody titer and affinity to LND was scarified and its sera were collected. The IgG fraction was isolated and purified by affinity chromatography on protein A column. The specificity of the purified antibody for LND was evaluated by indirect competitive ELISA using dexamethasone as a competitor as it is used with LND in a combination therapy.

**Conclusions:**

The high affinity of the antibody (IC_50_ = 10 ng/mL) will be useful in the development of an immunoassay system for the determination of plasma LND concentrations. Current research is going to optimize the assay conditions and validate the procedures for the routine application in clinical laboratories.

## Background

Cancer is one of the greatest health concerns, as it is the principal cause of mortality among men and women worldwide; it accounted for 7.4 million deaths (around 13% of all deaths each year). Deaths from cancer worldwide are projected to continue rising, with an estimated 12 million deaths in 2030 [1]. Multiple myeloma (MM) is a B-cell malignancy characterized by proliferation of monotypic plasma cells. It is the second most common hematological malignancy; approximately 20,000 cases of MM have been diagnosed in 2007 [2].

The hallmark of MM is the production of a homogeneous immunoglobulin fraction, called myeloma protein, by the malignant plasma cells [3]. Pathologic bone damage is the most characteristic feature of MM and is caused by the production of osteoclastic factors by the malignant plasma cells. Bone pain is the predominant presenting symptoms, but other symptoms such as anemia, hypercalcemia, renal insufficiency, neuropathy, and spinal cord compression may be present at the time of diagnosis. The classical triad of symptoms is plasmacytosis (> 30% of plasma cells in the bone marrow), myeloma protein either in the urine or blood, and lytic bone lesions [3,4]. MM is ultimately fatal with most patients relapsing after an initial response to the conventional chemotherapy. Autologous stem cell transplantation and melphalan have prolonged overall survival rates in patient populations, but most individuals eventually succumb to a refractory form of the disease.

In the 1990s, thalidomide (Thalomid®, Celgene Corporation) was used empirically in treatment of MM based on its antiangiogenic activity and clinical activity in refractory or relapsed myeloma [5]. However, thalidomide has significant and dose-limiting somnolence, constipation, neuropathy, and teratogenicity [6]. These toxic effects promoted the search for more potent but less toxic thalidomide derivatives [7]. Lenalidomide (LND) is a potent novel thalidomide analog which demonstrated remarkable clinical activity against myeloma cells [8-12] via a multiple-pathways mechanism [7,13-17]. The strong evidences-based clinical success of LND in patients has led to its recent approval by US-FDA under the trade name of Revlimid® capsules by Celgene Corporation [18].

Lenalidomide has a more improved side effects profile than its parent compound thalidomide, nevertheless, it causes some dose-dependent side effects such as thrombocytopenia, venous thromboembolism, and myelosuppression [19,20]. These side effects can be managed by combination therapy and/or careful dose adjustment [21,22]. Because LND is primarily excreted via kidneys, patients with renal insufficiency or failure must be dose adjusted to prevent the exacerbation of its myelosuppressive effects [9,16]. Because of the structural relation of LND to thalidomide, a teratogenic effect can not ruled out, thus effective contraception must be used by female patients [23,24]. Furthermore, studies showed large inter-individual pharmacokinetic variability with concentration–toxicity relationship [25]. For these reasons, a risk management, monitoring blood counts, and therapeutic drug monitoring are required to achieve the highest therapeutic benefits of LND and prevent its fatal complications [8,26,27]. Nevertheless, the therapeutic profile of LND is anticipated to encourage the development of new pharmaceutical preparations for LND. As a consequence, there is an increasing demand for proper analytical technologies for determination of pharmacokinetic parameters in bioequivalence studies for LND, as well as in its therapeutic monitoring.

Extensive literature survey showed that there were only two methods for the determination of LND in plasma [28,29]. These two methods involved liquid chromatography-coupled with mass spectrometric detectors. These two methods offered adequate sensitivities; however they employed the expensive mass detectors that are not available in most laboratories, and involved laborious liquid-liquid sample extraction procedures that negatively affected the accuracy of the results. Furthermore, these methods, as relied on sequential samples analysis, are not suited for screening of large number of specimens. Accordingly, the development of a new alternative analytical technology for the determination of LND in plasma with adequate sensitivity, improved simplicity, lower cost, and higher throughput is urgently needed.

Immunoassays are preferable in the field of clinical analysis because of their applicability for a wide range of analytes, high-throughput, low cost, convenience for screening of a large number samples, and their specificity for the analyte of interest even in multi-component complex sample matrix such as plasma [30]. The antibody is the most important key reagent in the development of any immunoassay system. As well, specificity of the antibody to the analyte of interest is the limiting factor in the validity of the assay. In order to establish a specific and sensitive immunoassay for LND, a specific antibody with high affinity for LND was required. The present study describes, for the first time, the synthesis of hapten for LND (modified LND with a 5-carbon glutaryl moiety as a spacer, and capable for direct linking to a protein) and preparation of a polyclonal antibody that can specifically recognizes LND with high affinity. This antibody will be useful for the establishment of a specific immunoassay for the determination of plasma LND concentrations. Optimization of the assay conditions and validation of the method for the routine application in clinical laboratories will be published elsewhere.

### Experimental

#### Apparatus

Microplate/cuvette reader (Spectramax M5, Molecular Devices, California, USA). Automatic microplate strip washer (MW-12A, Bio-Medical Electronics Co. Ltd., Shenzhen, China). EM-36N microtube shaker (Taitec, Japan). Biofuge Pico centrifuge (Heraeus Instruments, Germany). Incubator (KARL KOLB, Scientific Technical Supplies, Dreieich, Germany). Microprocessor laboratory pH meter (Mettler-Toledo International Inc., Zürich, Switzerland). Direct reading balance (XB 120A, Precisa Instruments Ltd., Switzerland). UV–vis Spectrophotometer (V-530, JASCO, Tokyo, Japan). Water purification (distillation apparatus, D-30938, GFL, Burgwedel, Germany). HPLC apparatus consisted of a Shimadzu system (Shimadzu Corporation, Kyoto, Japan) equipped with LC-10AD VP pump with FCV-10AL VP low pressure flow control valve, SCL-10A VP system controller, Rheodyne-7725 injection valve with variable loops, SPD-10A VP UV-visible detector, and RF-10A XL fluorescence detector. The system control and data acquisition are performed by Shimadzu CLASS-VP software, version 5.032 (Shimadzu Corporation, Kyoto, Japan). Mass spectrometric spectrum was conducted at the Department of Pharmaceutical Chemistry, College of Pharmacy, King Saud University, Saudi Arabia on Agilent Ion Trap 6320 LC/MS (Agilent Technologies, Saugus, MA, USA. NMR Spectra were scanned in DMSO-d6 on a Bruker NMR spectrophotometer operating at 500 MHz for ^1^H and 125.76 MHz for ^13^C at the research center, College of Pharmacy, King Saud University, Saudi Arabia. Chemical shifts were expressed in δ-values (ppm) relative to TMS as an internal standard. D_2_O was added to confirm the exchangeable protons

## Materials

Lenalidomide (LND) was obtained from LC Laboratories (Woburn, USA). Horseradish peroxidase labeled goat anti-rabbit IgG (HRP-IgG), bovine serum albumin (BSA), 2,4,6-trinitrobenzene sulfonic acid, 1-ethyl-3-(3-dimethylaminopropyl) carbodiimide hydrochloride (EDC), 3,3`,5,5`-Tetramethylbenzidine (TMB) peroxidase substrate, glutaric anhydride, and Freund's adjuvants (complete and incomplete) were purchased from Sigma Chemical Co. (St. Louis, MO, USA). Keyhole limpet hemocyanin (KLH) was purchased from Novabiochem Co. (La Jolla, CA, USA). BCA protein assay kit was purchased from Pierce Chemical Co., Rockford, USA). ELISA high-binding microwell plates were a product of Corning/Costar, Inc. (Cambridge, MA, USA). Dialysis tubes were from Sigma Chemical Co., St. Louis, MO, USA). All other chemicals and solvents used throughout the work were of analytical grade.

### Procedures

#### Synthesis of hapten (N-Glutrayl Lenalidomide; G-LND)

Lenalidomide (130 mg, 0.5 mmole) was added to glutaric anhydride (57 mg, 0.5 mmole) dissolved in 10 mL of benzene. The reaction was allowed to proceed under reflux for 24 hours. The reaction was monitored by HPLC system to confirm the formation of N-glutaryl LND. The chromatographic conditions were; reversed phase column (Nucleosil C_8_, 150 × 4.6 mm, 5 μm), isocratic elution by a mobile phase consisted of 20% acetonitrile containing 0.1% trifluoroacetic acid, and the flow rate was set at 1 mL/min. The eluted peaks were detected by UV detector at 254 nm. After completing the reaction, the *N*-glutaryl LND (G-LND) was purified by crystallization from ethanol. The purity of the product was confirmed by HPLC system. The chemical structure of the purified G-LND was confirmed by mass, ^1^H-NMR, and 13 C spectrometric techniques.

#### Preparation of LND protein conjugates

Fifty milligrams of G-LND (hapten) was dissolved in dimethylformamide. To this solution, 100 mg of EDC was added, and the pH was rapidly adjusted to 5–5.5 with 0.01 M HCl. After 5 min, the protein (BSA and KLH) solution (5 mg/mL) was added. Protein solutions were prepared by dissolving 50 mg of each of BSA and KLH in 5 mL of 50 mM phosphate buffer (PB) of pH 7.2. After addition of the protein solution, the pH of the reaction mixture was rapidly adjusted to 6.4 and maintained constant for 90 min. The reaction was left overnight in the dark at 4°C. The residual unreacted small LND hapten molecules were removed from the LND-protein conjugates by dialysis. The degree of conjugation of LND to each of BSA and KLH was then estimated by protein assay and UV spectral analysis [31].

#### Immunization of animals, preparation and purification of anti-LND antibody

Four rabbits were subjected to the immunization by LND-KLH conjugate. The immunization protocol, and monitoring the immune response of the animals have been conducted according to the procedures described by Darwish *et al.*[32]. The affinities of the raised antibodies to LND were determined by a competitive ELISA described by Darwish *et al.*[32]. The antibody that showed the highest affinity to LND was purified by affinity chromatography on protein A column.

## Results and discussion

### Synthesis and structure confirmation of LND Hapten (*N*-Glutaryl-LND)

**Figure 1:**
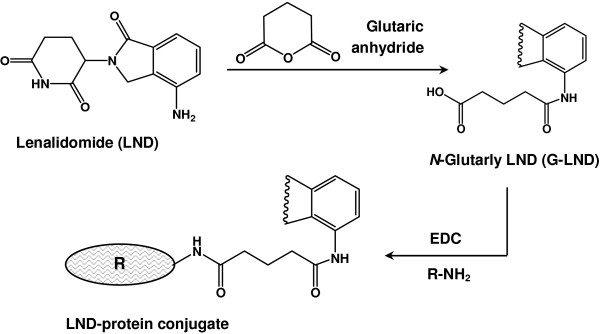
**Preparation of LND-protein conjugates.** EDC is ethyl-3-(3-dimethylaminopropyl) carbodiimide hydrochloride, and R−NH_2_ is the BSA and KLH proteins.

**Figure 2:**
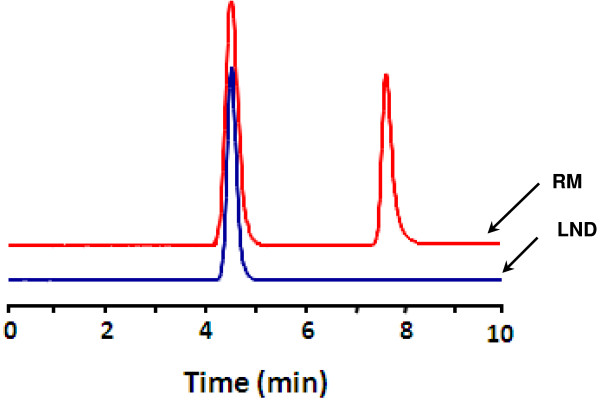
**Chromatogram of standard LND and its reaction mixture (RM) with glutaric anhydride.** Chromatographic conditions are: reversed phase column (Nucleosil C_8_, 150 × 4.6 mm, 5 μm), isocratic elution by a mobile phase consisted of 20% acetonitrile containing 0.1% trifluoroacetic acid, the flow rate was set at 1 mL/min, the detection was UV at 254 nm.

**Figure 3:**
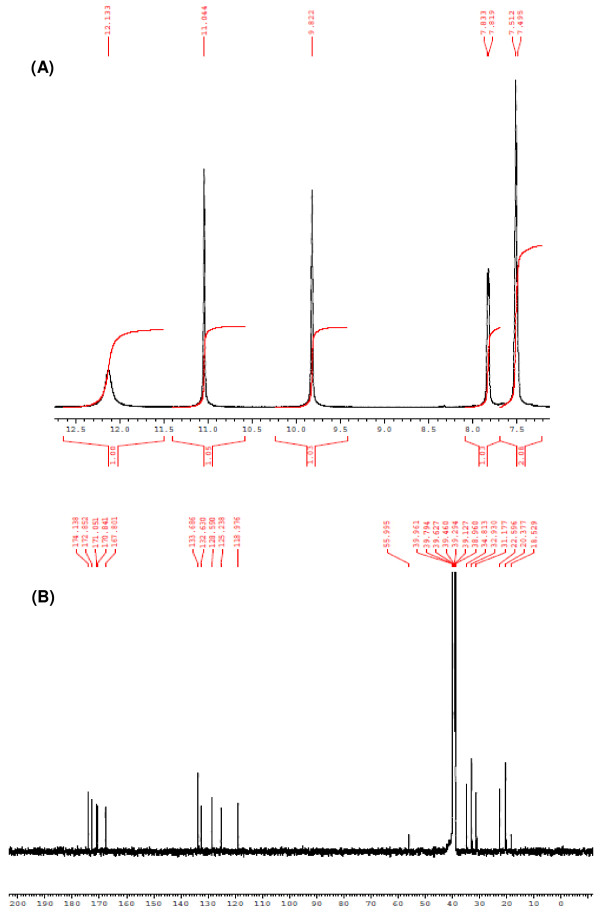
^**1**^**H-NMR (A) and**^**13**^**C (B) spectra of glutaryl-LND derivative.** NMR Spectra were scanned in DMSO-d6 on a Bruker NMR spectrometer operating at 500 MHz for ^1^H and 125.76 MHz for ^13^C. Chemical shifts are expressed in δ-values (ppm) relative to TMS as an internal standard. D_2_O was added to confirm the exchangeable protons.

Since LND is small molecule, it is not naturally immunogenic, and hence it does not elicit an immune response unless coupled with some macromolecules such as proteins. It was, therefore, required to modify LND for coupling with carrier protein so as to make a stable LND-protein conjugate. LND has a primary aromatic amino group via which LND could be directly conjugated with proteins by a well-established procedure of diazotization of the aniline group and conjugation to the tyrosine residues of the proteins [33]. However, it was reported that the introducing of a “spacer group” between the drug molecule and the carrier protein usually increases the specificity of the produced antibody [34]. Therefore the introducing a glutaryl moiety into the LND structure via its aniline group was attempted. This spacer (5 carbon atoms) was anticipated to be adequately spaced from the LND epitopic moiety [34]. Glutaryl residue was introduced into LND by reaction with glutaric anhydride (Figure 1), and as expected, two peaks appeared in the chromatogram of the reaction mixture (Figure 2). The firstly eluted peak showed retention time similar to that of LND (4.5 min) indicating that this peak corresponded to the unreacted LND. The second peak (at retention time of 7.8 min) was thought to be glutaryl-LND derivatives. LC/MS spectrometry for this peak gave a molecular weight of 372 indicating that it was the glutaryl derivative of LND. After completing the reaction, the product was purified and its structure was confirmed by ^1^H-NMR and ^13^C spectrometry (Figure 3).

The ^1^H NMR spectrum (DMSO-*d*_*6*_) of the product is given in Figure 3A. δ 1.81-1.84 (m, 2H, -CH_2_-CH_2_-COOH of glutaryl moiety) 2.2-2.6(m, 4H, -CH_2_-CH_2_-of piperidine ring), 3.4 (t, 4H,-CH_2_-CH_2_-CH_2_-COOH), 4.32-4.38, 4.35-4.4 (dd, *J*=32Hz, -CH_2_-N- of pyrrolidone ring), 5.16 (t, 1H, *J*=9, -CH of piperidine ring), 7.4-7.8 (m, 3H, ArH), 9.8 (s, 1H, NH, of the amide side chain, D_2_O exchangeable), 11.04 (s, 1H, NH of piperidine ring, D_2_O exchangeable), 12.13 (s, 1H, -COOH, D_2_O exchangeable). The figure depicts the aromatic protons part of the^1^H NMR spectrum and the three D_2_O exchangeable protons of the synthesized compound (two NH and one COOH protons).

The ^13^C spectrum of the product is given in Figure 3B: δ 18.5 (−CH_2_-CH_2_-COOH of glutaryl moiety), 20.3 (−CH_2_-CH_2_-C=O of piperidine ring), 22.5 (−CH_2_-CH_2_-C=O of piperidine ring), 31.1 (−CH_2_-CH_2_-COOH), 32.9 (−CH_2_-CONH of glutaryl moiety), 34.8 (−CH_2_-N- of pyrrolidone ring), 55.9 (−CH of piperidine ring), 118.9, 125.2, 128.5-132.6, 133.6 (−CH Aromatic), 167.8 (−CH-C=O of piperidine ring), 171 (−CH2-C=O of piperidine ring), 172.8 (−COOH), 174.1 (C=O amide of glutaryl moiety). Upon comparing these data with those of the starting material (standard LND), it was clear the difference between LND and the synthesized compound at the upfield region of the spectra indicating the aliphatic part of the side chain as well as the appearance of new two signals downfield indicating the presence of C=O carboxylic acid and C=O amide.

### Preparation and characterization of LND-Protein conjugates

The hapten (G-LND) was used for its linking to carrier proteins. Bovine serum albumin (BSA) and keyhole limpet hemocyanin (KLH) proteins were selected as carrier proteins because they are rich in lysine residues that can couple with the carboxylic moiety of the G-LND [35]. Linking of G-LND with BSA and KLH proteins was achieved by carbodiimide method [36] as illustrated in Figure 1.

**Figure 4:**
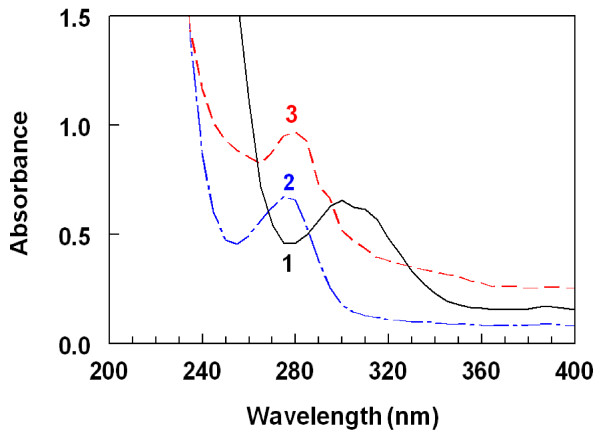
**Absorption spectra of G-LND (50 μg/mL; 1) and equal concentrations (0.5 mg/mL) of BSA (2) and LND-BSA conjugate (3).** Concentration of LND-BSA conjugate was determined as BSA protein. Concentrations were prepared in phosphate buffered saline.

In order to ascertain the conjugation and estimate the extent to which G-LND was conjugated to the proteins, spectral analysis for the LND-protein conjugates were conducted. Under the same conditions, the UV absorption spectra of the LND-protein conjugates, the unconjugated hapten (G-LND), and the proteins were recorded. The spectra that have been generated in case of BSA conjugate are given in Figure 4, and similar results were obtained with KLH protein. Obviously, the absorbances of the LND-protein conjugates were higher than those of equal concentrations of unconjugated proteins at their maximum absorption peaks (280 nm). These hyperchromic effects were evident for the successful conjugation of LND with both BSA and KLH. The degree of conjugation of drug with both BSA and KLH was determined spectrophotometrically according to the method described by Habeeb [31]. The percentages of LND residues in LND-BSA and LND-KLH conjugates were found to be 36.52 and 24.85%, respectively.

### Preparation and characterization of anti-LND antibody

**Figure 5:**
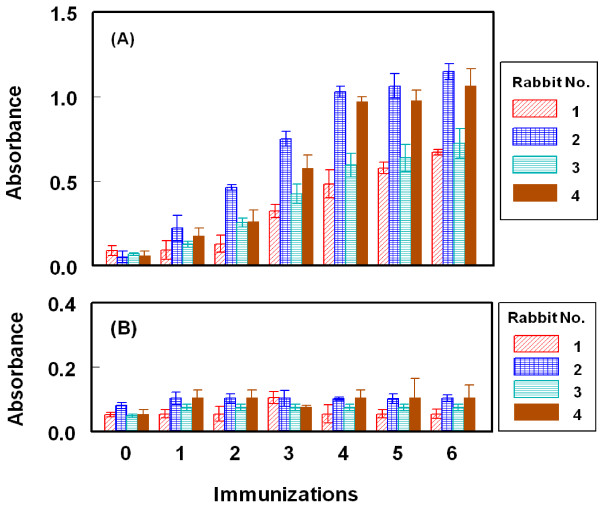
**Monitoring the immune response of rabbits immunized with LND-KLH.** Microwell plates were coated with LND-BSA (panel **A**) and BSA (panel **B**). Antiserum samples after different consecutive immunizations were allowed to bind to the immobilized antigen (BSA and LND-BSA). Detailed procedures and signals were generated as described by Darwish *et al.*[32].

**Figure 6:**
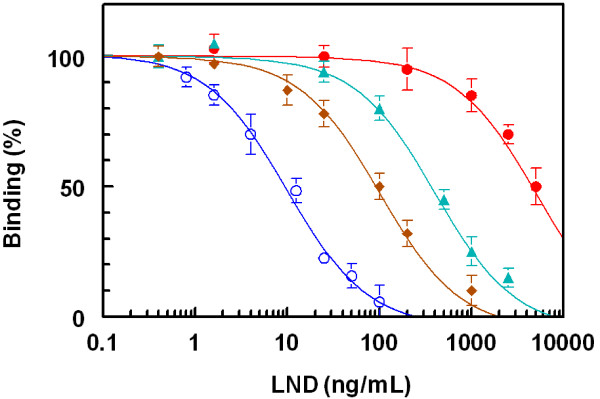
**Affinity of antisera from rabbits No. 1 (●), No. 2 (**○**), No. 3 (▲), and No.4 (♦) for LND. LND-BSA was coated onto the microwell plates.** Antiserum samples were mixed with LND and transferred into the microwells. Competitive binding reaction was allowed to proceed and the signals were generated as described by Darwish *et al.*[32].

Because of the high immunogenicity of KLH protein [36], LND-KLH conjugate was selected as immunogen for immunization of animals and LND-BSA conjugate was selected for immobilization onto the microwell plates in the ELISA. To monitor the progress of the immune response of the rabbits and confirm that they were sufficiently immunized, serum samples were collected from the rabbits on the fourth to seventh days after each immunization, and were analyzed by the direct ELISA [32]. As shown in Figure 5A, the titers of the antisera (indicated by the absorbance values) increased with the repetitive immunizations. As well, it was observed that the reactivity of the produced antibodies to the immobilized protein (BSA) was comparable to that before immunization (Figure 5B). These data indicated the specificity of the raised antibodies to the LND residues in the immobilized LND-BSA, but not BSA molecules. The measured small absorbance values were attributed to the nonspecific binding. Although, all rabbits responded well to the repetitive immunizations, however in order to select the most appropriate animal for scarifying and collecting the total sera, the affinity of these antisera from all animals were checked by competitive ELISA as described above. As shown in Figure 6, the serum from rabbit No. 2 shows the highest affinity (lowest IC_50_) for LND, therefore this rabbit was scarified and its total sera were collected as crude anti-LND antibody, and it was purified by affinity chromatography on protein A column.

### Purification of anti-LND antibody

**Figure 7:**
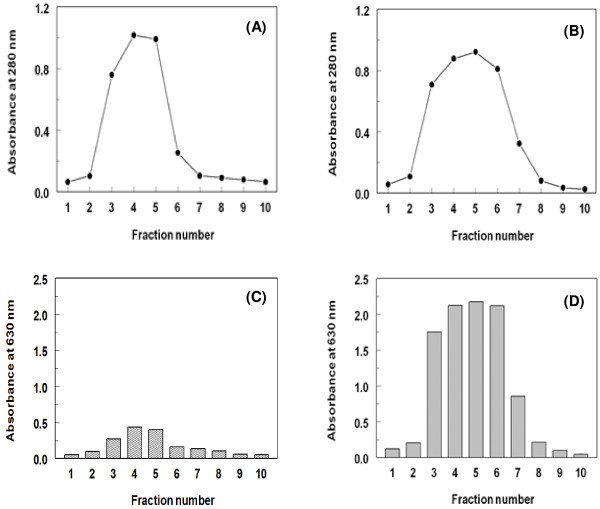
**Purification of anti-LND antibody from rabbit serum by protein A column affinity chromatography.** (**A**) and (**B**) are the binding and elution profiles of protein A column chromatography, respectively. (**C**) and (**D**) are immunoreactivity of the fractions eluted from protein A column to the immobilized LND-BSA, determined by the direct ELISA.

The immunoglobulin fraction of the antibody specific to LND was isolated from the crude serum sample of rabbit No. 2 by precipitation with saturated ammonium sulphate solution followed by purification on protein A column using affinity chromatography. Protein A was considered in this study based on its efficient binding to wide range of immunoglobulins, and the easy elution of these antibodies by mild chromatographic conditions [37]. For purification of the antibody, serum sample was applied onto the protein A column that has been equilibrated with the immunoglobulin-binding buffer (glycinate buffer of pH 8.7), and the eluent was fractionated. The elution of the unbound UV-absorbing species was monitored by measuring the absorbances of the fractions at 280 nm. After complete elution of the UV absorbing unbound species (Figure 7A), the immunoglobulin-eluting buffer (citrate buffer of pH 3) was applied to elute the immunoglobulin fraction that has been bound to the protein A (Figure 7B). To confirm the binding of the anti-LND antibody to the protein A by glycinate buffer, and its elution with citrate buffer, the immunoreactivity of each fraction was tested by the direct ELISA using LND-BSA conjugate as an immobilized antigen. The very weak immunoreactivity of the fractions obtained while applying glycinate buffer indicated that most of the immunoglobulins were bound to the protein A (Figure 7C). As well, the high immunoreactivity of fractions obtained while applying the citrate buffer indicated the efficient elution of the immunoreactive immunoglobulins by the citrate buffer (Figure 7D), and most of the bound immunoglobulins were eluted in the fractions No. 3–7. These fractions were collected, pooled and dialyzed against PBS to remove the salts of buffer solution. The protein concentration of the purified antibody was determined by BCA kit according to the procedures recommended by the manufacturer.

### Immunoreactivity and specificity of the purified anti-LND antibody

**Figure 8:**
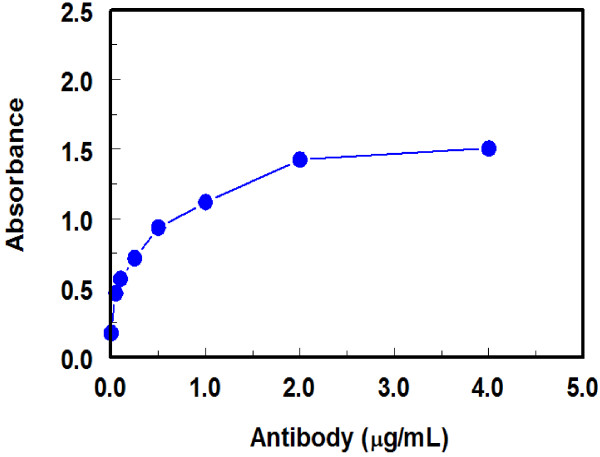
Titration of the purified anti-LND antibody versus immobilized LND-BSA conjugate.

In order to ascertain that the immunoreactivity of the anti-LND antibody did not affected by buffer changes during its purification by protein A column chromatography, titration of anti-LND versus a fixed concentration of the solid phase LND-BSA was carried. It was found that the binding, indicated by absorbances, increases as the antibody concentration increases (Figure 8). This proved that the immunoreactivity of the antibody has not been affected during its purification. In addition, this titration gave the optimum limiting concentration of the antibody that can be used for effective competition between free LND (as competitor in the test sample) and immobilized LND (LND-BSA coated onto the plate wells), The limited (not saturating) antibody concentration that gave 1–1.5 absorbance unit (in the direct ELISA) was 1 μg/mL (Figure 8). This concentration was used in assessment of the specificity of the anti-LND antibody by the competitive ELISA.

The specificity of the antibody was determined by carrying out the competitive assay using dexamethasone that is administered in combined therapy with LND [38], as a competitor. It was found that dexamethasone does not show any immunoreactivity with the anti-LND antibody. In conclusion, this antibody will be useful for the establishment of a specific immunoassay system for accurate determination of plasma concentrations of LND. Optimization of the assay conditions, validation of the method, and clinical applications are currently going on in our laboratory, and the results will be published elsewhere.

## Conclusions

The present study described the synthesis of N-glutaryl LND as a hapten, and the preparation of a highly specific polyclonal antibody against LND. The antibody recognizes LND with high affinity. The high specificity and affinity of the produced antibody enables the development of highly specific and sensitive immunoassay system for the accurate determination of LND in plasma. The method is expected to contribute to the therapeutic monitoring and pharmacokinetic studies of LND.

## Abbreviations

MM: Multiple myeloma; LND: Lenalidomide; G-LND: N-glutaryl lenalidomide; ECD: 1-ethyl-3-(3-dimethylaminopropyl) carbodiimide hydrochloride; BSA: Bovine serum albumin; LND-BSA: Lenalidomide conjugate of bovine serum albumin; KLH: Keyhole limpt hemocyanin; LND-KLH: Lenalidomide conjugate of keyhole limpt hemocyanin; ELISA: Enzyme-linked immunosorbent assay; US-FDA: United States Food and Drug Administration; HRP-IgG: Horseradish peroxidase labeled goat anti-rabbit immunoglobulin IgG; TMB: 3,3`,5,5`-tetramethylbenzidine; PB: Phosphate buffer; PBS: Phosphate buffered saline; PBS-T: Phosphate buffered saline containing 0.05% Tween-20.

## Competing interests

The authors declare that they have no competing interests.

## Authors’ contributions

IAD proposed the subject, designed the study, participated in the results discussion and revised the manuscript. NZA participated in the study design, results discussion, and preparing the manuscript. RMA synthesized the hapten, conducted the preparation of the protein conjugates, immunization and purification of the antibody. TEE conduct the spectroscopic analysis of the hapten and interpret the spectral data. All authors read and approved the final manuscript.
